# An Underactuated Adaptive Microspines Gripper for Rough Wall

**DOI:** 10.3390/biomimetics8010039

**Published:** 2023-01-16

**Authors:** Xinxin Li, Wenqing Chen, Xiaosong Li, Xin Hou, Qian Zhao, Yonggang Meng, Yu Tian

**Affiliations:** Department of Mechanical Engineering, Tsinghua University, Beijing 100084, China

**Keywords:** microspines gripper, bionic mechanism, interfacial attachment, under-actuated mechanism

## Abstract

Wall attachment has great potential in a broad range of applications such as robotic grasping, transfer printing, and asteroid sampling. Herein, a new type of underactuated bionic microspines gripper is proposed to attach to an irregular, rough wall. Experimental results revealed that the gripper, profiting from its flexible structure and underactuated linkage mechanism, is capable of adapting submillimeter scale roughness to centimeter scale geometry irregularity in both normal and tangential attachment. The rigid-flexible coupling simulation analysis validated that the rough adaptation was achieved by the passive deformation of the zigzag flexible structure, while the centimeter-scale irregularity adaptation come from the underactuated design. The attachment test of a spine confirmed that a 5 mm sliding distance of the spine tip on the fine brick wall promises a saturated tangential attachment force, which can guide the stiffness design of flexible structure and parameter selection of underactuated linkage. Furthermore, the developed microspines gripper was successfully demonstrated to grasp irregular rocks, tree trunks, and granite plates. This work presents a generally applicable and dexterous passive adaption design to achieve rough wall attachment for flat and curved objects, which promotes the understanding and application of wall attachment.

## 1. Introduction

A number of creatures, from small insects such as ants and cockroaches to large animals such as cats and eagles, are able to attach themselves to the rough walls through their claws. They realize attachment by interlocking between their microspines at their claw ends and the rough peak on the rough surface [1,2,3,4,5,6]. Dai et al. [7] studied the tangential attachment behavior between the claws of the free-walking beetle and sandpaper, with emphasis on the relationship between the dimension of the claw tip and the asperity Attachment by spine/surface interlocking shows excellent adaptability to dusty, rough surfaces, which are common in nature and the human living environment.

Based on this effective attachment by spine/surface interlock in nature, a unique technology, namely microspines, was developed [8]. A microspine consists of one or more steel hooks embedded in a rigid frame with a compliant suspension system made of elastic flexures or spring elements. By arraying tens or hundreds of microspines, a large load can be supported and shared between many attachment points. Since each microspine has its own flexible structure, it can stretch and drag independently to find a suitable asperity to anchor. Microspines enable gravity-independent mobility on rocky surfaces, including cliff faces, lava tubes (including the ceiling), and in microgravity environments such as the surfaces of near-Earth asteroids [9,10,11].

Microspines technology has been utilized in climbing robots [8,12,13,14,15,16] and grippers [17,18,19,20]. Asbeck et al. [8,13,21] developed the first vertical wall climbing robot, SpinyBotII, by using microspines. The robot consists of six feet with ten independently movable microspines, which are made of shape deposition manufacturing (SDM) [22,23]. SDM enables the mixing of flexible materials and hard materials into a whole structure. Asbeck also proposed the criteria for the design of the flexible structure of microspines and established the spring damping model to analyze the compliant performance [24,25]. The microspines gripper developed by Parness et al. [9] has 16 microspines boxes distributed circumferentially, with a total number of microspines exceeding 250. Each microspines box can move independently. Meanwhile, each microspine in each claw box can also move independently. This multi-level flexible design enables it to achieve millimeter-scale roughness adaptation. On rough rock, the microspines gripper can achieve an attachment force of over 100 N in all directions. Wang et al. [20] proposed a new microspines gripper, which was applied to the Robosimian humanoid robot of the Jet Propulsion Laboratory to realize rock climbing. The microspines array in the gripper can independently move up and down. There are 19 microspines per square centimeter, greatly increasing the density of claws. A pulley differential system achieves a uniform load share. Homoplastically, Wang et al. [26] applied the microspines to a multi-finger manipulator. Ruotolo et al. [27] proposed multi-finger manipulators for large climbing robots that use jamming to regulate the state of the gripper to achieve a uniform load share.

In this paper, a new type of underactuated bionic microspines grippers is proposed to attach to an irregular rough wall. The gripper employs a flexible structure to achieve a submillimeter- to millimeter-scale roughness adaptation and utilizes an underactuated linkage mechanism to achieve a millimeter- to centimeter-scale level geometry adaptation. By designing the flexible structure of the spine, the stiffness design criteria of the flexible structure are proposed. The stiffness of the bionic mechanism is optimized by finite element analysis. The design of the spines, compliant structure, and underactuated linkage are introduced in detail. The rigid-flexible coupling simulation analysis model is established to help with the design of the mechanism. Finally, the developed microspines gripper is tested and verified for its good performance on surface roughness and irregularity adaptation.

## 2. Materials and Methods

### 2.1. Design of the Microspines Grippers

#### 2.1.1. Overall Design of the Microspines Grippers

**Figure 1:**
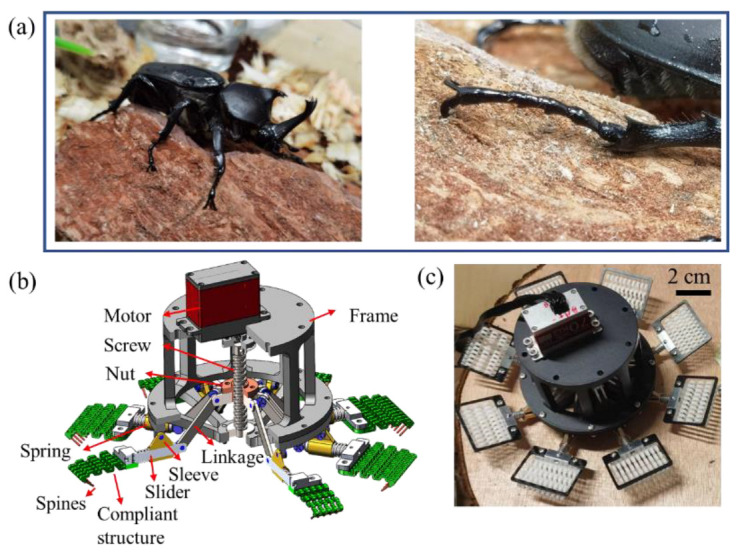
Design and fabrication of microspines gripper. (**a**) Bionic design motivation from Rhinoceros beetle (Coleoptera: *Phileurus didymus*) attachment. (**b**) CAD (Computer-aided design) model of the microspines gripper. (**c**) The microspines gripper prototype.

The inspiration for the microspines grippers is from the claw of the rhinoceros beetle (Coleoptera: *Phileurus didymus*). Figure 1a shows the rhinoceros beetle attach to the rough rock surface by its claw interlocking with the rough peak. With the adaptability of its foot joints, it can adapt to the rough wall very well. The overall scheme and real object of the microspines gripper are shown in Figure 1b,c. The opposing grasping scheme is used to achieve the attachment load in all directions. The microspines are arranged in a circular array of eight microspines boxes on a gripper, which is driven by a lead screw nut mechanism. The microspines are bonded to the flexible structure; 10 microspines form a microspine unit that is fixedly connected to the sliding rod, and the microspine unit can slide along the sleeve axis. A compression spring is arranged between the sliding rod and the sleeve to constrain the redundant degrees of freedom of the underactuated mechanism. The end of the sliding rod is connected with the link through a hinge to form a rotation pair. The link is connected to the lead screw nut through a hinge to form a rotation pair. These parts form a single gripper. The mechanical motion pair constitutes an underactuated linkage, and the other flexible structures are the passively adaptive key components of the microspines. The whole microspines is driven by only one motor, which can realize attachment and detachment at the same time.

#### 2.1.2. Spine Design

The structure of the spine is important for attachment performance. In theory, the smaller the radius of the spine tip, the stronger the adaptability to the surface with a smaller roughness. However, the smaller the spine tip radius the lower its strength, and the maximum adhesion that a single spine can withstand will also be limited. Otherwise, the tip is prone to wear, plastic deformation, or even fracture, which makes the microspines bear more adhesion to the same object. On the premise of ensuring the adhesion, it is also very important to improve the adhesion of a single spine as much as possible. The main failure modes of the spine tip after attachment include plastic deformation, excessive wear, or even fracture due to the insufficient strength of the claw tip, and skipping due to the insufficient strength of the rough peak. The latter mainly depends on the properties of the wall. For sandpaper with 40 mesh, the rough peak is weak and cannot bear excessive load. For cement bricks, floor tiles, and other surfaces, their rough peak strength is relatively high, and generally there is no fracture, so the microspine is often the failure in this situation.

**Figure 2:**
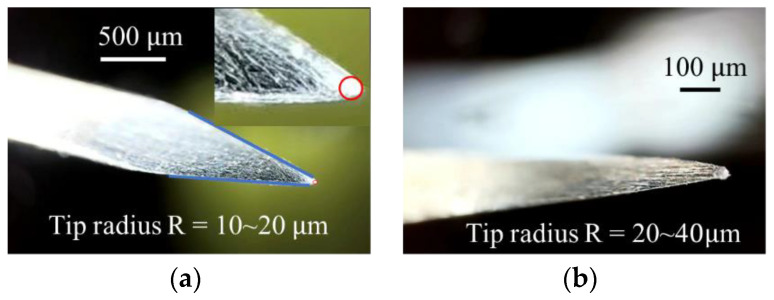
The structure of the spine. (**a**) Before wear. (**b**) After wear.

In order to increase the strength and wear resistance of the spines, the spines are made of stainless steel and chrome-plated on the surface. The mechanism is designed with an overload protection mechanism. The spine used in this paper is a commercial needle. The needle tip is shaped as a triangular cone. The diameter of the needle handle is about 0.7 mm, the cone angle of the needle tip is about 24°, and the radius of the needle tip is 10 μm~20 μm. When the spine grasps the wall, it will slide for a certain distance before meeting the appropriate rough peak. This process will also cause the tip of the microspine to wear. Figure 2 shows the comparison of the needle tip before and after wear. It can be found that the needle tip is worn, resulting in a larger radius of curvature of the needle tip, about 40 μm~60 μm. This will reduce the ability of the worn claw to lock the rough peak, so the performance of the whole microspines will also be reduced.

#### 2.1.3. Compliant Structure Design

The flexible structure is mainly used to improve the adaptability of the microspines to the rough wall surface, so that the load of different microspines is evenly distributed as far as possible. They jointly bear the normal and tangential adhesion, so as to avoid the damage of a single microspine due to overload. On the basis of Asbeck et al. [28], this section considers the design requirements of flexible structures as follows:

(1) Distributing the overall tangential and normal force evenly to avoid damaging individual spines and avoid “peeling” failures, in which stress concentrations cause a local failure that then propagates across the array;
(2) Provides enough independence between adjacent spines such that each spine can find and settle on its own asperity without influencing or being influenced by its neighbors;
(3) Maintaining the orientation of each spine so that it does not slip off its asperity as loads are increased;
(4) Accommodating errors in alignment between the spine array and the wall and accommodating local curvature in the wall surface.

In order to meet the requirements of the application of microspines in the actual scene, researchers have tried a variety of flexible structure preparation schemes. Asbeck et al. [25] used shape deposition manufacturing (SDM) technology to make a flexible structure with nonlinear stiffness by multi-component molding and established a spring bar model to analyze its mechanical behavior, which can better meet the design requirements mentioned above. However, the flexible structure made by SDM has the disadvantages of low strength and high cost. Parness et al. [9,10,28,29,30] made a variety of flexible structures with the laser cutting method in view of asteroid attachment and sampling requirements, taking into account the reliability of the exoplanet environment and flexible structures. Liu et al. [31] used selective laser sintering (SLS) rapid prototyping to make a zigzag flexible structure by imitating the characteristics of arthropod adaptation.

In this study, a similar flexible structure is made by Stereolithography Appearance (SLA), and the flexible structure is optimized as required. The printing material uses the Future 8200 Pro material produced by the Future Factory Company. The elastic modulus of this material is about 2.6 GPa, the tensile strength is about 60 MPa, and the thermal deformation temperature reaches 58 °C. It has good toughness and high printing quality. In this device, 10 flexible microspines can be arrayed to form a single microspine foot.

In the process of determining the design dimensions of flexible structures, three structures are initially selected in this paper, and the stiffness matrix is calculated by finite element simulation. The stiffness matrix represents the relationship between the load applied to the needle tip and its displacement:

(1) $$F=K\delta =\left[\begin{array}{c} F_{s}\\ F_{n}\\ M_{\theta } \end{array}\right]=\left[\begin{array}{ccc} K_{\mathrm{xx}} & K_{\mathrm{xy}} & K_{x\theta }\\ K_{\mathrm{yx}} & K_{\mathrm{yy}} & K_{y\theta }\\ K_{\theta x} & K_{\theta y} & K_{\theta \theta } \end{array}\right]\left[\begin{array}{c} \delta_{s}\\ \delta_{n}\\ \delta_{\theta } \end{array}\right]$$

where *F* is the force acting on the needle tip, *K* is the stiffness matrix, *δ* is needle tip displacement.

The coefficients of the stiffness matrix shall meet:

(1) *K*_xx_ should be moderate, so that the flexible structure can not only meet the requirement that the deformation is greater than the average rough peak spacing of the wall surface in the horizontal tension direction but also withstand large loads;
(2) *K*_yy_ should be smaller to meet the adaptability of microspine to the millimeter level of wall geometry;
(3) *K*_xy_ should be less than zero. At the attachment stage, after anchoring the rough peak, give the remaining microspine a force to continue to approach the wall, so that as many microspines as possible can contact the wall well and anchor the appropriate rough peak.

The finite element analysis is carried out in Comsol Multiphysics 6.0. The displacement of the unit length is applied to the needle tip, respectively, and the force acting on the needle tip is calculated. The corresponding stiffness matrix coefficients can be calculated using Equation (1). The stiffness matrix is considered a linear system, and the stiffness matrix is a diagonal symmetric matrix. The stiffness matrix is obtained as:

(2) $$K=\left[\begin{array}{ccc} 1.5\;N/\mathrm{mm} & -0.42\;N/\mathrm{mm} & 5\;N/\mathrm{rad}\\ -0.42\;N/\mathrm{mm} & 0.46\;N/\mathrm{mm} & -6.4\;N/\mathrm{rad}\\ 5\;N/\mathrm{rad} & -6.4\;N/\mathrm{rad} & 11.4\;N\cdot \mathrm{mm}/\mathrm{rad} \end{array}\right]$$

**Figure 3:**
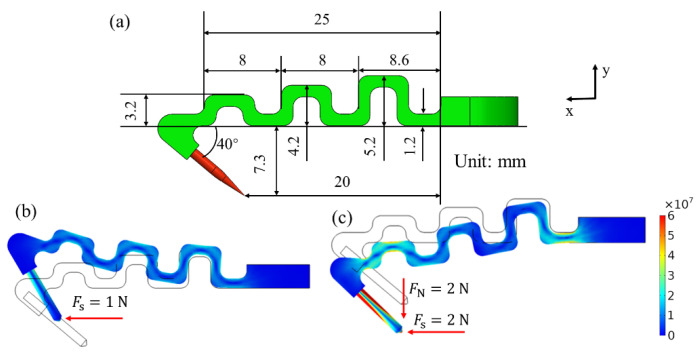
Analysis diagram of flexible structure size, grasping load and pulling load. (**a**) Original dimension of flexible structure. (**b**) Possible stress state during grasping. (**c**) Possible stress state after grasping.

Figure 3 shows the size of the flexible structure and the stress nephogram under load. The zigzag assembly of flexible structures can achieve both normal and tangential flexibility, and its stiffness coefficient can be adjusted by the labelled sizes. In the process of grasping, the tip of the microspine slides horizontally on the wall to find a suitable rough peak. When it encounters a rough peak, it bears the force in the horizontal direction. At this time, the possible stress state is shown in Figure 3. It can be found that the microspines’ angle deflects at this time. This will make it easier for the microspines to jump over the rough peak. This mechanism is called automatic filtering. This automatic filtering will filter out those unreliable rough peaks before grasping, so as to make the microspine find enough reliable rough peaks to attach.

In addition, after being subjected to the tangential force, the microspine has a tendency to move away from the wall, which shows that after a microspine is anchored. It will drive other microspines to approach the wall. In the process of lifting and loading, the tip of the microspine will bear the normal force from the wall, and the tangential force will also increase. At this time, it is found that the force in these two directions will make the angle of the microspine return to its initial state as far as possible, which will improve the contact reliability between the tip of the microspine and the rough peak after grasping. In addition, the maximum stress of the flexible structure printed by Stereolithography Appearance (SLA) on the result surface of the stress nephogram in the figure is about 50 MPa, which occurs at the root of the connection. However, the stress generally does not exceed this load in actual use. If overload occurs in some special cases, flexible structure fracture may still occur, but individual flexible structure fracture will not have a significant impact on the overall adhesion performance.

### 2.2. Rigid-Flexible Coupling Simulation Analysis

In order to further analyze the grasping performance and influence parameters of the microspines gripper, this section will analyze the dynamics and elasticity of the coupling mechanism based on the Comsol Multiphysics 6.0 software platform. At the same time, considering the influence of an underactuated device and flexible structure parameters on the adhesion performance, the rigid-flexible coupling simulation model of the microspines will be established and the quasi-static analysis will be carried out. On this basis, the anisotropic attachment effect of the microspines on the large, flat, rough surface is analyzed.

**Figure 4:**
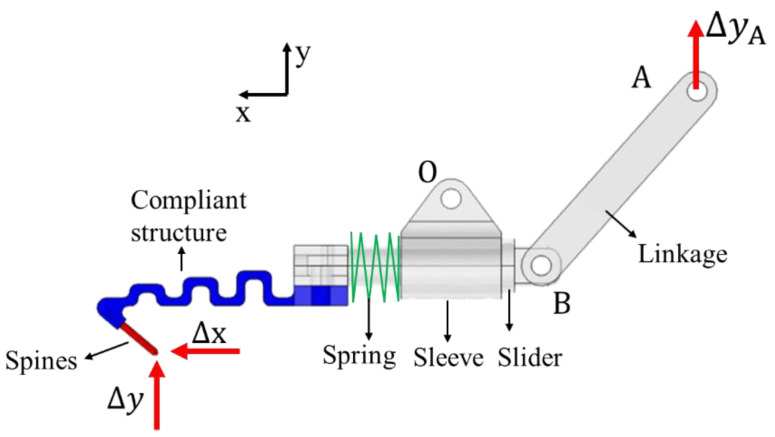
Rigid-flexible coupling simulation model of spines single foot.

Considering that the eight microspines units are the same and distributed circumferentially. Only one prong is taken as the research object to analyze various working conditions, and then the attachment behavior of the entire microspines is obtained. Figure 4 shows the rigid-flexible coupling simulation model. Its CAD model is built in Solidworks, and some unnecessary parts, such as bolts and pin shafts, are simplified and then imported into Comsol Multiphysics.

The built-in multi-body dynamics (MBD) physical field is used for rigid-flexible coupling analysis, in which the flexible structure and jaw are set as elastic bodies and the rest of the components are set as rigid bodies. Point A is the rotation center in link AB, and the incremental displacement of A simulates the action of the lead screw nut on the link. Then the driving force can be calculated. The link AB and slider are set as hinge joints at point B, ignoring the friction effect. A prismatic joint is set between the slider and the sleeve. A spring damper is added to simulate the effect of the compression spring. The relative displacement of the constraint is controlled between 0~5 mm. The sleeve can hinge with the frame by constraining the position of the point O as its rotation center and only releasing its rotational freedom around the z axis. By setting the corresponding boundary conditions at the tip of the needle, it can be used to simulate a variety of behaviors during actuation, grasping, and loading.

### 2.3. Test of Microspines Gripper

**Figure 5:**
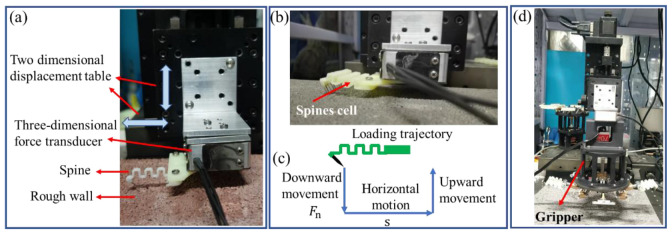
Experimental testing apparatus. (**a**) Attachment forces test of a spine. (**b**) Attachment forces test of a spine cell. (**c**) Loading trajectory of attachment test. (**d**) Attachment forces test of a gripper.

In order to fully understand the performance of the microspines, a test bench, as shown in Figure 5, was built to test the single microspine, microspines unit, and gripper at three levels. A two-dimensional motion table is used to realize horizontal and vertical motion. The microspines unit is installed on the displacement table through a three-dimensional force sensor, which can monitor the three-way force received by the needle tip during the attachment process. The attached wall is placed on the platform and fixed with limit screws. The whole experimental device is built on the optical platform. The test process for single microspine and microspine modules is basically the same. Additionally, the module can be replaced directly during installation. The test process is as shown in Figure 5b. The microspines unit is first pressed to the wall by the vertical displacement table. The Labview upper computer detects the force in real time. The vertical movement will stop for 10 s and start a horizontal movement when the normal force at the contact between the needle tip and the wall reaches the set value *F*_n_. The displacement table pulls the microspines unit for a specified distance s. After a pause of 10 s, the vertical displacement table drives the microspine module to completely disengage from the wall.

When testing the gripper, install it under the force sensor. In each experiment, the gripper first lifted the single foot of the microspine. The vertical displacement table moves downward to make the jackscrew of the movable microspines contact the wall surface. Then the normal contact force can automatically determine whether it is in contact or not. After the contact is completed, the gripper is used for actuating and grasping. Finally, the vertical displacement table moves upward, pulls the gripper away from the wall, and measures the peak adhesion.

## 3. Results and Discussion

### 3.1. Results of Rigid-Flexible Coupling Analysys

**Figure 6:**
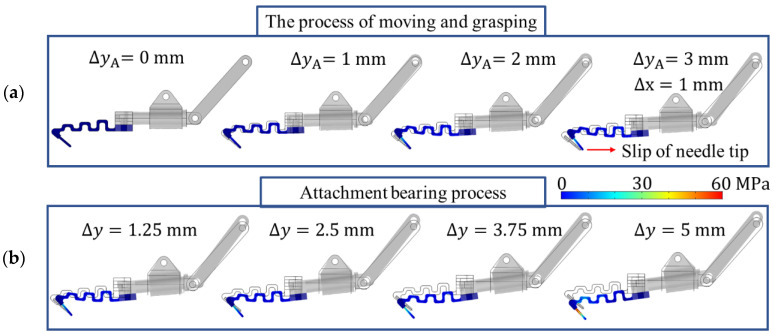
Action sequence and stress nephogram of single foot of microspine in the process of grasping and attaching. (**a**) Actuate the grasping process. (**b**) Attachment loading process.

Figure 6 illustrates the process of tip moving and grasping. First, consider that the microspine tip has already contacted the micro-rough peak. By fixing the microspine tip, set an upward displacement at point A of the link to move at a uniform speed Δy = 3 mm, simulating the actuating and grasping processes. When the needle tip horizontal force *F*_s_ reaches 10 N, the tip begins to slip distance Δx and rebalance. The average spacing of rough peaks generally does not exceed 2 mm, so the constraint is 0 ≤ Δx ≤ 2 mm. Then, the grasping process is completed, and the microspines begins to bear load. The bearing process is simulated by applying the displacement Δy = 5 mm in the negative direction of the *y*-axis on the tip of the needle.

**Figure 7:**
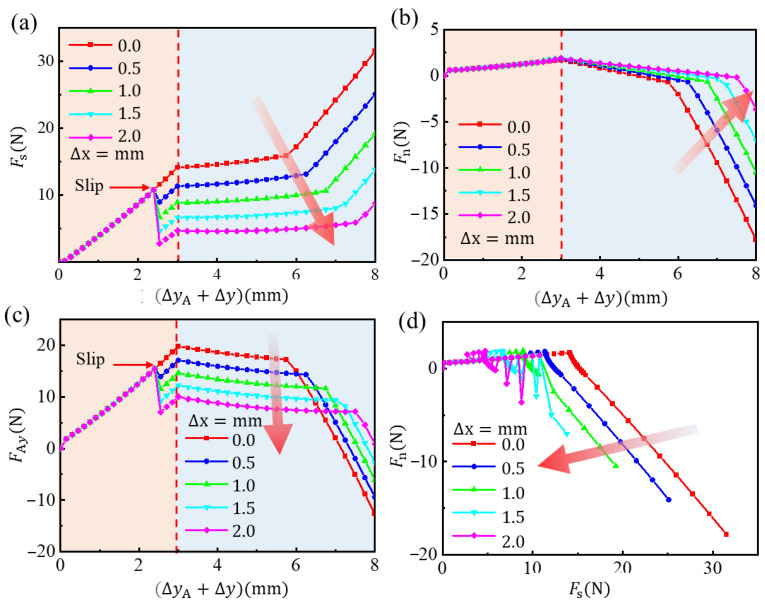
The forces of the single foot of the spines in the process of grasping and attaching. (**a**) Tangential force of needle tip. (**b**) Normal force of needle tip. (**c**) Normal force at hinge point A. (**d**) Stress diagram of needle tip in force space.

Figure 6 shows the motion diagram of the microspine unit at each time when Δx = 1 mm and the stress cloud diagram of the flexible structure. Figure 7 analyzes the stress in this process. It can be found that in the process of actuating loading, the microspine tip is anchored to the wall. Additionally, the flexible structure undergoes flexible deformation under the action of the underactuated linkage mechanism. The normal force and tangential force between the needle tip and the wall gradually increase. The ratio of the two is less than 0.15, so it will slide on the surface when encountering an inappropriate rough peak. This process can enable the microspine to find a suitable rough peak for attachment. Due to the rough peak that may be cracked, the microspine tip may jump and slip when s reaches 10 N. Meanwhile, the sliding will cause a sudden reduction in tangential force while the normal force is basically unchanged. This behavior can also ensure that the needle tip will not slide too far, providing favorable support for secondary anchoring of rough peaks. Figure 7 shows that the driving force required by the single foot of the microspine does not exceed 30 N. That is, the driving force required by the whole microspines does not exceed 300 N, so the motor used can fully meet the requirements.

**Figure 8:**
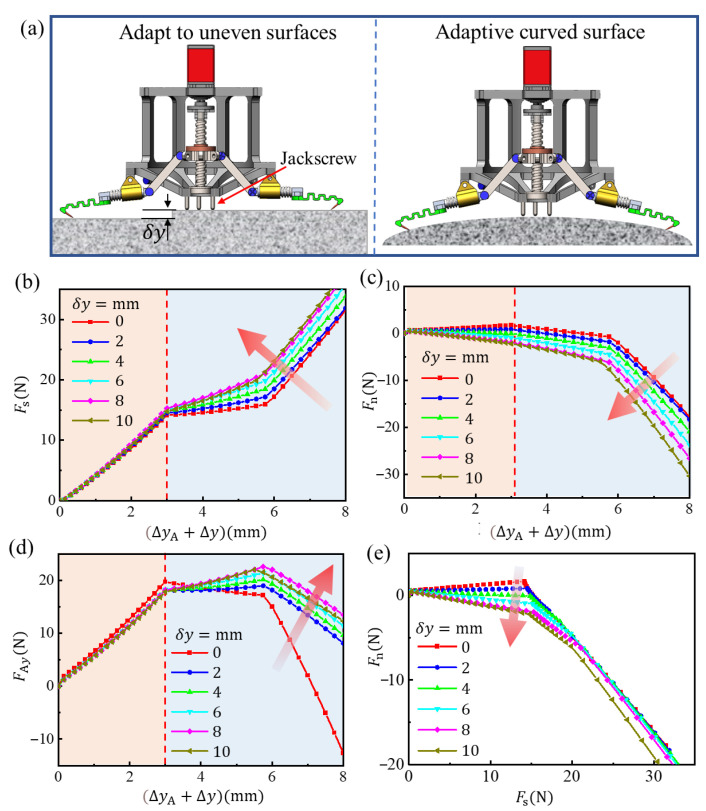
The microspines gripper adapts to uneven and curved surfaces. (**a**) Diagrammatic sketch. (**b**) Tangential force of needle tip. (**c**) Normal force of needle tip. (**d**) Normal force at hinge point A. (**e**) Stress diagram of needle tip in force space.

The impact of an uneven or irregular curved surface on attachment is also very important. As shown in Figure 8a, whether it is adapted to an uneven wall or a curved surface, it can be uniformly considered as the attachment performance of the needle tip when anchoring the rough peak at different normal positions. At this time, the simulation will be carried out according to the following process: given 0 ≤ *δ*y ≤ 10 mm, the state of a single foot of microspine can be obtained. This is taken as the initial state to calculate the process of the action, grasp and attachment of the single foot of the microspine. As before, the upward displacement is still set at point A of the link to move uniformly Δy = 3 mm and complete the actuation and attachment process. After that, apply the displacement Δy_A_ = 5 mm in the negative direction of the *y*-axis on the tip to simulate the loading process.

The results are as shown in Figure 8b–d. In the actuation grasping stage, the tangential force (*x*-axis direction) when the microspine contacts the wall surface has little difference. It is worth noting that the normal force (*y*-axis direction) may change from the positive pressure to the desorption force, which is different when adapting to the plane bulge and curved surface. If it is suitable for the plane bulge, the normal force becomes negative, which means that the microspine may detach from the wall surface before anchoring the rough peak, which is not allowed. However, if the claw is attached to a curved surface, the claw tip will still remain in contact. When *δ*y is about 4 mm, the normal contact force between the tip and the wall is close to 0 N, which is a very instructive result. If the plane is grasped in this state, the tip will not have too much friction with the wall when it slides on the wall to find an effective attachment point, which can reduce the wear of the tip.

Compared with the horizontal state, the flexible structure in this state is more stressed along its axial tension, and the stiffness in this direction is greater, so the adhesive bearing capacity is stronger. Therefore, we can adjust the height of the jackscrew in Figure 8 to a proper position so that the microspine can attach in this state when grasping the plane. Further, with the increase of *δ*y, the adhesive bearing capacity will also increase. When the microspines grasp the surface, the biggest difference is that the tangential friction between the tip and the wall also contributes to the overall adhesion. Therefore, the attachment ability of the microspines to curved objects will be enhanced to a certain extent, and the load safety line shown in Figure 8e will be inclined clockwise to the normal angle of the wall, which is widely studied in the research of gecko-like dry adhesive grippers and will not be described in depth here.

### 3.2. Performance of Microspines Gripper

**Figure 9:**
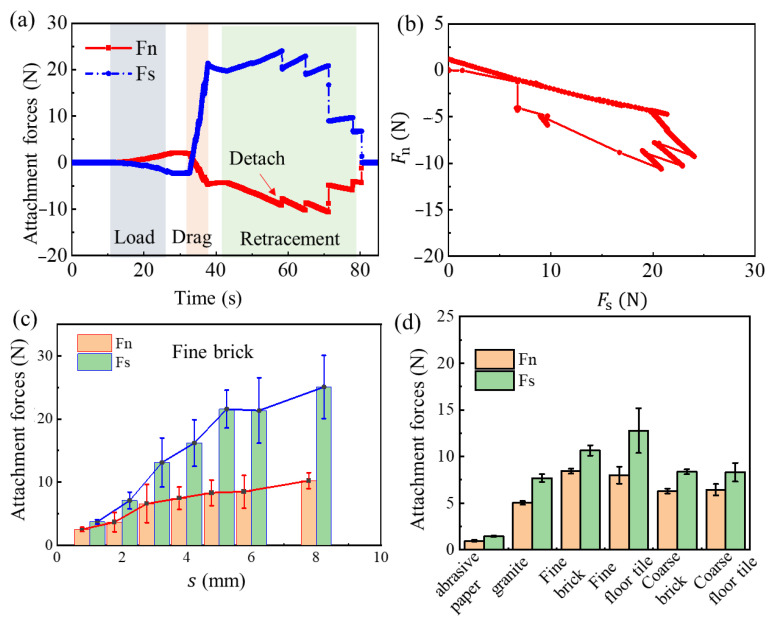
Performance of microspines Gripper. (**a**) Attachment forces test of the spines unit. (**b**) Attachment forces of the spines unit in force space. (**c**) The relationship between attachment forces and slip distance *s*. (**d**) Attachment forces of microspines cell on six surfaces.

Figure 9 shows the test results of the microspine unit. During the downforce test, the microspine contacts the wall until the normal contact force reaches 2 N. The normal force and tangential force in this process are in opposite directions, which indicates that the normal deformation of the flexible structure will cause the tangential contraction. The normal force increases twice with the downforce distance. After the pause, drag the distance from one end horizontally, and then the tangential force rises rapidly and reverses; the normal force also decreases and reverses. At this time, most of the microspines in the prong unit have achieved good adhesion.

In the process of withdrawal desorption, the normal adhesion is negative. With the further lifting of the displacement table, the microspines begin to detach, which is reflected in the sudden fluctuation of the force curve. It is worth noting that the normal adhesion is not the largest when the claw stingers detach for the first time but the largest later. This shows that in the actual grasping process, the initial few claw stingers detached will not necessarily lead to the complete failure of the attached claws. This verifies the robustness of the microspines in the grasping process from the side. In this process, tangential adhesion presents a relatively stable trend. Until the number of microspines desorbed increases, finally complete desorption is achieved. The results in the force space show that the microspines will detach when they reach the load safety line. Generally speaking, as long as the microspine does not break, it will detach according to the wall adhesiveness criteria analyzed in Section 2.1.

Among the factors that affect the performance of the spines’ element, the horizontal towing distance and the flexible structure occupy the main positions. As shown in Figure 9c, the adhesion gradually increases with the increment of the towing distance. The increment of adhesion becomes gentle after the towing distance reaches 5 mm. Therefore, when the microspines are actually attached, the horizontal towing distance should be about 5 mm. If the adhesion is too small, it is not enough. If the adhesion is too large, it is easy to overload and damage the microspine, and it is also easy to cause excessive wear of the microspine tip.

Figure 9d shows the test results of the microspine unit on different wall surfaces. It can be found that fine cement and fine floor tiles are the best, followed by coarse cement bricks and coarse floor tiles; granite plates are inferior; and sandpaper is the worst. This is mainly due to the difference in morphology between these surfaces. The sandpaper surface rough peak is relatively gentle, and there are relatively few attachment points, which can be found from the previous probability model.

### 3.3. Grasping Demonstration of Microspines Gripper

**Figure 10:**
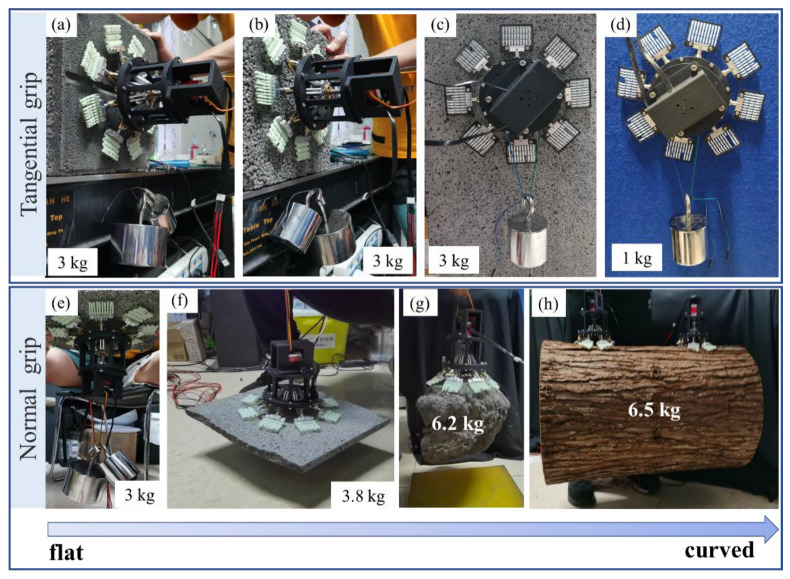
Tangential and normal grasping demonstration of microspines gripper on different surfaces. (**a**) Fine brick. (**b**) Coarse brick. (**c**) Granite. (**d**) Fabric. (**e**) Coarse brick. (**f**) Granite. (**g**) Rock. (**h**) Trunk.

The microspines gripper can adapt to a variety of surfaces in the actual wall attachment. It can also achieve normal attachment and tangential attachment at the same time. Figure 10a–d shows its performance in tangential adhesion. Without considering the self-weight of 500 g, it can easily adhere more than 3 kg on the wall of coarse cement, fine cement bricks, and granite. On the fabric wall, due to the limitation of the fabric’s surface strength, its tangential adhesion can also reach more than 1 kg. the performance of the microspines in normal attachment. It can attach more than 3 kg to the wall of coarse and fine cement, coarse and fine cement bricks, and granite.

Figure 10e–h shows the performance of microspines gripper in normal adhesion. The grasping movies about granite plane, irregular rock, and tree trunk can be saw in Supplementary Material in S1, S2 and S3, respectively. It is worth mentioning that the microspines grab a deformed rock weighing 6.2 kg in Figure 10g, reflecting their centimeter-scale wall geometric adaptability. Of course, the microspines can also be used to grasp a spherical object with a rough surface. Figure 10h shows that two microspines are used to grasp a tree trunk model, and each microspines uses only four microspine elements. The weight of the trunk model is more than 6 kg, and the roughness of the bark on the surface is more than 5 mm. This grasping case reflects the extraordinary adaptability of the microspines.

## 4. Conclusions

In conclusion, based on the bionic attachment of insects, an underactuated bionic microspines gripper is proposed. The gripper adopts an axisymmetric design and uses a bionic flexible structure and an underactuated linkage mechanism to realize passive adaptation to the wall roughness and geometric shape. A drive motor is used to realize passive adaptation to the surface roughness of millimeter to centimeter level. It uses 80 microspines to achieve a normal adhesion of more than 60 N and a tangential adhesion of more than 30 N. The rigid-flexible coupling model with a flexible structure and an underactuated linkage mechanism can effectively improve the adhesion performance of the microspines under various working conditions, providing theoretical guidance for the design of the microspines.

## Data Availability

The data that support the findings of this study are available upon reasonable request from the authors.

## Supplementary Materials

The following supporting information can be downloaded at: https://www.mdpi.com/article/10.3390/biomimetics8010039/s1.
